# Low-Complexity Saliency Detection Algorithm for Fast Perceptual Video Coding

**DOI:** 10.1155/2013/293681

**Published:** 2013-12-23

**Authors:** Pengyu Liu, Kebin Jia

**Affiliations:** School of Electronic Information & Control Engineering, Beijing University of Technology, Beijing 100124, China

## Abstract

A low-complexity saliency detection algorithm for perceptual video coding is proposed; low-level encoding information is adopted as the characteristics of visual perception analysis. Firstly, this algorithm employs motion vector (MV) to extract temporal saliency region through fast MV noise filtering and translational MV checking procedure. Secondly, spatial saliency region is detected based on optimal prediction mode distributions in I-frame and P-frame. Then, it combines the spatiotemporal saliency detection results to define the video region of interest (VROI). The simulation results validate that the proposed algorithm can avoid a large amount of computation work in the visual perception characteristics analysis processing compared with other existing algorithms; it also has better performance in saliency detection for videos and can realize fast saliency detection. It can be used as a part of the video standard codec at medium-to-low bit-rates or combined with other algorithms in fast video coding.

## 1. Introduction

With the rapid developments of multimedia information processing and communication technology, video encoding has become the basic core technology of digital television, video conferencing, mobile media, 3D video coding, and so forth. During the past decades, in order to obtain video codec with low complexity, high quality, and high compression ratio, various technologies have been proposed for fast video coding [1].

Studies have shown that human visual system (HVS) is sensitive to the video scene perception and assigns different visual importance to different regions [2, 3]. Researchers have used advantages of visual attention in various multimedia processing applications such as image retargeting and video coding; the researching of saliency detection model for perceptual video coding is a hot topic. One of the key processing steps is to perform low-complexity calculations and obtain region of interest (ROI) in accordance with visual perception characteristics timely and effectively.

Up to now, saliency detection algorithms are widely used in extracting ROI in videos for various multimedia processing applications [4–8]. Moving zone detection technique for pixel precision is able to detect the moving foreground area, but its complexity in calculation makes it not applicable in real-time encoding. Liu et al., [6] proposed the moving zone detection algorithm, but the algorithm mainly employs motion vector information, making it even not applicable in effective detection on moving object in global motion zone. Yuming et al., in [7] a video saliency detection algorithm based on feature contrast is proposed, but the computational efficiency needs to be further improved.

Those saliency detection algorithms mentioned previously are facing a common problem: they are not only time-consuming but computation-consuming as well. Those algorithms do not pay more consideration to the effect of the real-time coding performance because of the additional computation for visual perception analysis. Complex saliency detection algorithm will increase the computational burden of video encoder, which is not conducive to the video coding standards in real-time multimedia communication application.

In this paper, a fast saliency detection algorithm based on low-level encoding information and HVS is proposed. In order to simplify the visual perception analysis progress, this algorithm correlates the encoding information in video bit-stream with the visual perception characteristics. The spatial and temporal saliency detection is carried out by means of MV. The prediction modes and other auxiliary coding information can save an amount of computing time in feature extraction for saliency detection. As almost no additional computation is increased to the video codec, the saliency detection computation complexity is lower compared with other existing algorithms. The saliency detection results are satisfied with the compared algorithms. So the proposed algorithm can reach the balance between the saliency detection accuracy and computational complexity.

This paper is organized as follows.  Section 2 is an overview of the proposed framework and describes in detail each one of its subsystems.  Section 3 gives the evaluation of the proposed algorithm, and in Section 4, some important conclusions are obtained and the further work is also introduced.

## 2. The Proposed Saliency Detection Algorithm

**Figure 1:**
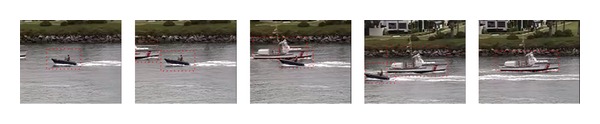
Tracking smooth movement targets (coastguard).

**Figure 2:**
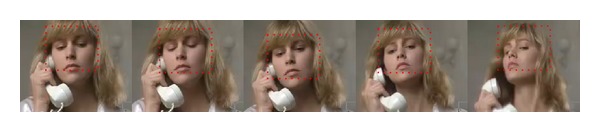
Interest region of foreground goal (Suize).

Motion is a highly salient feature which grabs one's attention and keeps it locked on important features and objects. Interest in motion perception has a long history and it can be considered as a relatively well established discipline [9]. Motion perception is one of the most important visual processing mechanisms. The visual information related to temporal motion would generate stronger response in HVS. HVS always pays more attention to the objects with smooth movement (as shown in Figure 1). Spatial contrast is the most basic visual processing mechanism in HVS; it is the prerequisite for HVS perception of spatial shape such as texture and object (as shown in Figure 2).

The response intensity of temporal motion visual information caused by HVS is larger compared with that of spatial motion visual information caused by HVS [10]. In the proposed algorithm, temporal saliency detection is performed firstly, and then spatial saliency detection is adopted in order to optimize the visual perception characteristics analysis results.

### 2.1. Temporal Saliency Analysis and Detection

**Figure 3:**
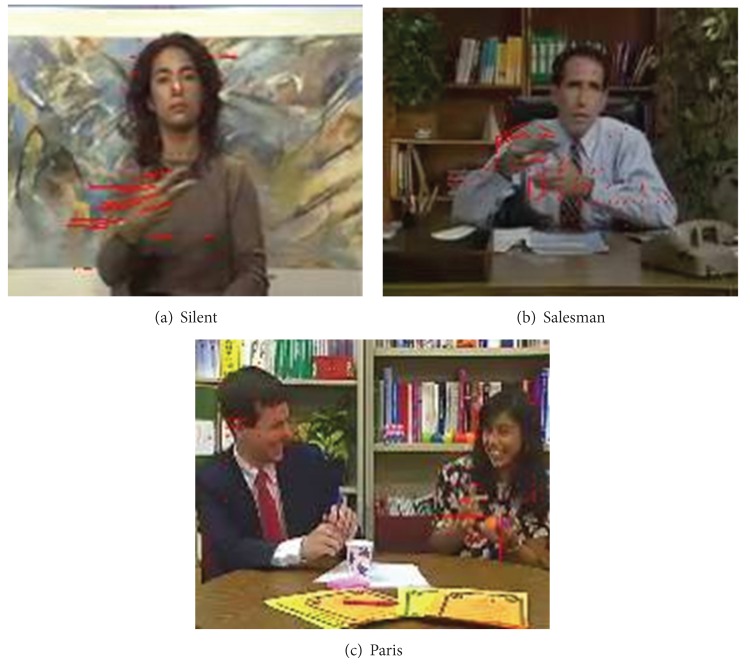
MV distribution in static background video frames.

As we know, moving objects always have larger MV in video frame. It can be found in Figure 3 that the coding regions with larger MV happen to be the ROI (such as head, face, shoulders, and arms); the coding regions with smaller MV or zero MV are always in the static background which could only arouse lower attention of HVS. To sum up, as a relatively high consistency exists between MV and visual attention, MV can be regarded as the temporal characteristics of visual perception. Many existing algorithms are used in order to get the motion feature for the motion saliency detection. These algorithms are only effective for videos with a static background.

In an ideal case, foreground moving object can generate nonzero MV. As there are no relative movements in the background region, it should produce zero MV in static background. However, in reality, nonzero MV noise would be generated randomly due to external change of illumination and internal change of video encoding parameters (such as change of quantization steps, motion search scheme, and rate distortion optimization algorithm). As a result, corresponding MV detection mechanism should be proposed to get rid of the interference of MV noise.

**Figure 4:**
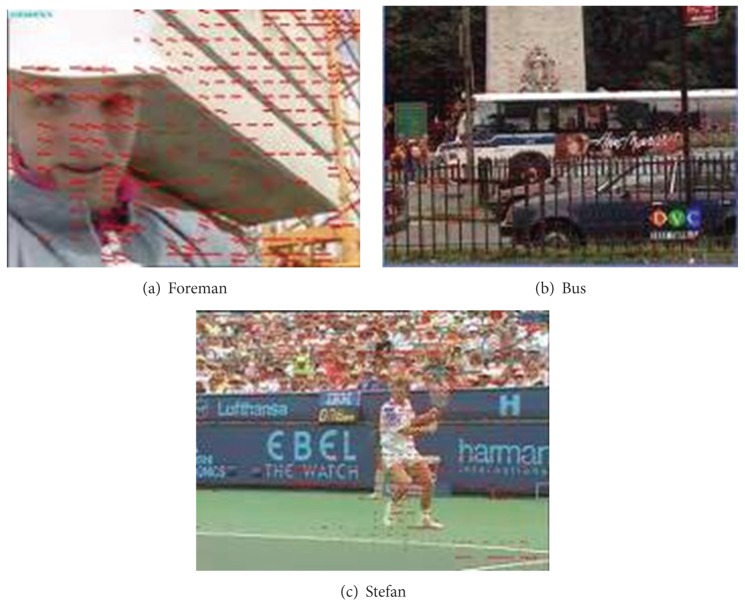
MV distribution in translation background video frames.

Besides the MV noise interfering in temporal saliency detection, the translation MV interfering from the background should be considered. As shown in Figure 4, in Foreman sequence, the foreground object is the human's head and shoulder. However, due to the rightward rotation of the camera, buildings on the right-hand side are moved into the scene, creating a globally distributed MV. In the same way, the horizontal displacement of camera causes obvious MV along the road side and the parked car in bus sequence. However, HVS is only interested in the moving bus on the road. Analogously, in Stefan sequence, the most interesting object is the tennis ball athlete which generated a lot of motion vector. But due to the moving of camera, there are amount of MV appeared on bleachers as well. Under these conditions with horizontal motion, the information of MV does not match the visual attention. Motion detection which is merely based on the size of MV can lead to errors in the temporal saliency judgment. As a result, corresponding MV detection mechanism needs to be formulated as well, in order to remove the interference caused by MV errors which are generated due to horizontal movement.

In order to improve the temporal detection accuracy, the MV noise filtering and the attenuating translation MV interference error should be added. At the same time, the complexity of processing procedure should be controlled strictly. Otherwise it will influence the real-time performance of the saliency detection algorithm.

#### 2.1.1. Filtering MV Noise

(*1) Basic Principle of MV Noise Filtering.* Compression coding uses the correlation between adjacent macro blocks to remove redundant information on spatial domain or temporal domain, in order to achieve a large amount of information with a small number of bits. In video coding framework based on blocks, the coded object is usually divided into several macro blocks or subblocks; then the macroblocks or subblocks that belong to an object should tend to have a similar motion vector or predictive coding mode and have similarity in structure [11]. Research shows that the moving objects in video sequence have the motion continuity and integrity. Motion continuity is reflected in there being a strong correlation between macro blocks in the current frame and the previous frame with corresponding position. Motion integrity reflects there existing great structural similarity between adjacent macro blocks in the same frame.

Therefore, based on the video sequence motion continuity and high temporal correlation, the MV of the macro block (MB) with the same position in the previous frame can provide very important prior information for the current MV of encoding MB.

**Figure 5:**
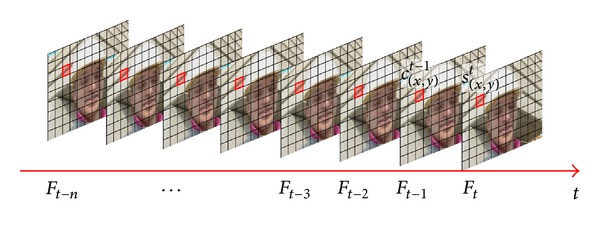
Schematic diagram of MBs with corresponding position in adjoining frames.

In Figure 5, *s*_(*x*,*y*)_^*t*^ represents the current coded MB, *t* represents the current encoded video frame, and (*x*, *y*) is the position coordinates. ${\vec{V}}_{s}$ is the MV of *s*_(*x*,*y*)_^*t*^. *c*_(*x*,*y*)_^*t*−1^ represents the MB in the previous frame with the same position coordinates as current coded MB and ${\vec{V}}_{c}$ is the MV of *c*_(*x*,*y*)_^*t*−1^.

Through using large amounts of test sequences and statistics based on the H.264/AVC standard (JM18.7), it can be found that ${\vec{V}}_{s}$ and ${\vec{V}}_{c}$ have a high correlation.

Take Akiyo sequence and Foreman sequence as representations for gentle motion and active motion of the two kinds of video sequences; the quantization parameter (QP) is set as 28 and 32, respectively, through using full search prediction method, statistics the joint probability $p(\vec{V}{}_{s}|{\vec{V}}_{c})$ of ${\vec{V}}_{s}$ and ${\vec{V}}_{c}$. The statistical results are shown in Table 1.

From Table 1 statistics data, it can be found that if ${\vec{V}}_{c}=0$, the probability of ${\vec{V}}_{s}=0$ is more than 60%. If ${\vec{V}}_{c}\neq 0$, the probability of ${\vec{V}}_{s}\neq 0$ and belong to the ${\vec{V}}_{c}$ (1 ± 10%) is nearly 80% for gentle motion sequence and more than 65% for active motion sequence. If ${\vec{V}}_{c}\neq 0$, the probability of ${\vec{V}}_{s}=0$ is less than 20%.

The simulation results show that ${\vec{V}}_{c}$ can be taken as an important basis for determining ${\vec{V}}_{s}$ being MV noise or not. As there exists strong motion continuity and relativity of the moving object in a video sequence, in order to reduce the error judgment rate, in this paper, based on the average MV of reference region in previous frame, the basic principle of MV noise filtering is proposed as follows.

**Figure 6:**
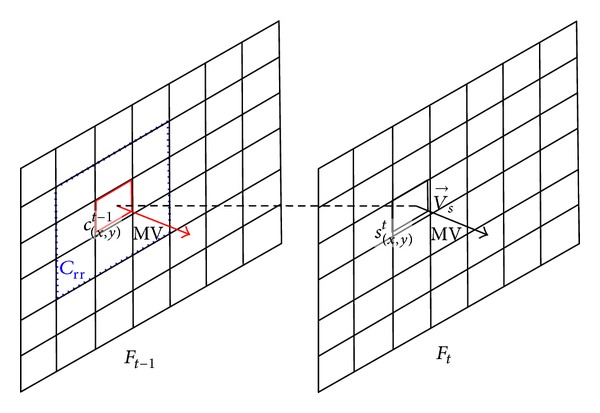
Schematic diagram of position relationship.

If ${\vec{V}}_{s}$ is generated in the current encoding MB *s*_(*x*,*y*)_^*t*^, there is a high probability that a MV with similar direction and size exists in the reference region *C*_rr_ of the corresponding position in the previous frame. If there is no MV in *C*_rr_, ${\vec{V}}_{s}$ should be treated as MV noise and be filtered out. So how to determine the reference area *C*_rr_ is the key factor affecting the MV filtering results.

(*2) Define Reference Region C*_*rr*_. As shown in Figure 6, *c*_(*x*,*y*)_^*t*−1^ is the MB which has the same position coordinates as MB *s*_(*x*,*y*)_^*t*^ in the previous frame. The rectangular area surrounded by dashed lines is defined as reference region *C*_rr_.

According to the direction of ${\vec{V}}_{s}$ (horizontal motion, vertical motion, and oblique motion), *C*_rr_ is determined as follows.

**Figure 7:**
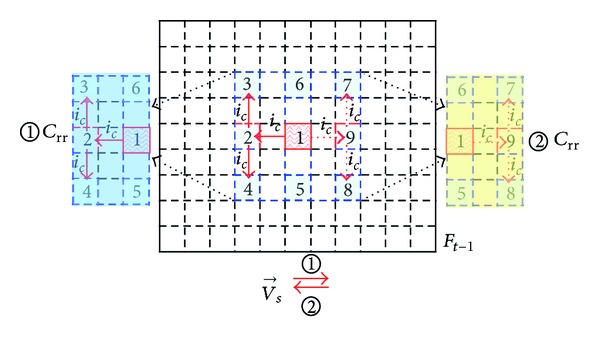
Schematic diagram of *C*_rr_ with horizontal motion.

*(i) C*_*rr*_ *with Horizontal Motion*. As shown in Figure 7, if one takes horizontal motion towards the right direction of ${\vec{V}}_{s}$ as an example, in the previous reference frame *F*_*t*−1_, find MB-1, which has the same position coordinate with the current encoding MB signed *c*(*x*, *y*).

Firstly, take MB-1 as the starting point, then perform horizontal motion of *i*_*c*_ macro blocks in the opposite direction of *v*_*s*_, and get MB-2 signed *c*(*x*, *y* − *i*_*c*_).

Secondly, centered at MB-2, make vertical extension of *i*_*c*_ macro blocks both upwards and downwards to obtain two vertical vertices, that is, MB-3 signed *c*(*x* − *i*_*c*_, *y* − *i*_*c*_) and MB-4 signed *c*(*x* + *i*_*c*_, *y* − *i*_*c*_).

Last, determine the rectangular reference region *①* and *C*_rr_ is designated by four macro blocks which are MB-3, MB-4, MB-5, and MB-6.

If ${\vec{V}}_{s}$ is a horizontal motion towards the left direction, use the same method to determine the rectangular reference region *②*; *C*_rr_ is designated and surrounded by four macro blocks which are MB-5, MB-6, MB-7, and MB-8.

In the above description, the position coordinates of MB-3 to MB-8 are given in Table 2.

In Table 2, *i*_*c*_ is defined as

(1) $$\begin{array}{c} i_{c}=\left[\frac{\left|\vec{V}{}_{sx}\right|}{w_{s}}+1\right]. \end{array}$$

$\left|\vec{V}{}_{sx}\right|$ denotes the MV magnitude of the horizontal direction ${\vec{V}}_{s}$. *w*_*s*_ denotes the width of the current coding block. [·] represents the round numbers calculation.

*(ii) C*_*rr*_ *with Vertical Motion*. If ${\vec{V}}_{s}$ make the vertical downward motion, the processing steps to determine reference region *③* *C*_rr_ are as follows.

Firstly, take MB-1 as the starting point, then perform vertical motion of *j*_*c*_ macro blocks in the opposite direction of ${\vec{V}}_{s}$ and get MB-2 signed *c*(*x* − *j*_*c*_, *y*).

Secondly, centered at MB-2, make horizontal extension of *j*_*c*_ macro blocks both leftwards and rightwards to obtain two horizontal vertices: MB-3 signed *c*(*x* − *j*_*c*_, *y* − *j*_*c*_) and MB-4 signed *c*(*x* − *j*_*c*_, *y* + *j*_*c*_).

**Figure 8:**
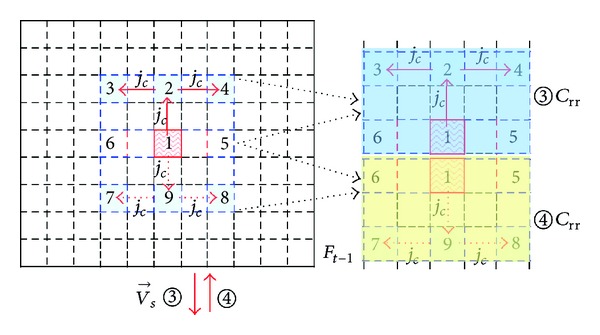
Schematic diagram of *C*_rr_ with vertical motion.

Last, determine the rectangular reference region *③*; *C*_rr_ is designated and surrounded by four macro blocks which are MB-3, MB-4, MB-5, and MB-6 (as shown in Figure 8).

If ${\vec{V}}_{s}$ make vertical upward motion, use the same method to determine the rectangular reference region *④*; *C*_rr_ is designated and surrounded by four macro blocks which are MB-5, MB-6, MB-7, and MB-8.

In the above description, the position coordinates of MB-3 to MB-8 are given in Table 3.

In Table 3, *j*_*c*_ is defined as

(2) $$\begin{array}{c} j_{c}=\left[\frac{\left|\vec{V}{}_{sy}\right|}{h_{s}}+1\right]. \end{array}$$

$\left|\vec{V}{}_{sy}\right|$ denotes the MV magnitude of the vertical direction *v*_*s*_. *h*_*s*_ denotes the height of the current coding block. [·] represent the round numbers calculation.

**Figure 9:**
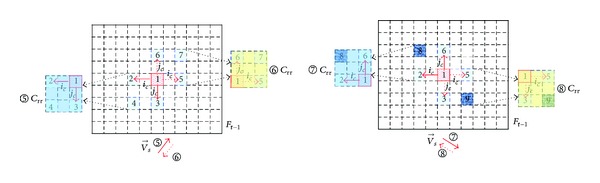
Schematic diagram of *C*_rr_ with oblique motion.

*(iii) C*_*rr*_ *with Oblique Motion*. If ${\vec{V}}_{s}$ make the oblique motion, determine the reference regions of *⑤* *C*_rr_, *⑥* *C*_rr_, *⑦* *C*_rr_, and *⑧* *C*_rr_ in the same way according to the different motion directions of ${\vec{V}}_{s}$. *C*_rr_ is given in Figure 9.

The position coordinates of MB-1 to MB-9 are given in Table 4.

In Table 4, *i*_*c*_ and *j*_*c*_ are defined as formulas (1) and (2).

In the proposed algorithm, reference region *C*_rr_ is not stationary, and the area and position of *C*_rr_ are changed adaptively according to the size and direction of ${\vec{V}}_{s}$.

(*3) The MV Noise Filtering Computational Formula.* Consider

(3) $$\begin{array}{c} T_{1}\left(x,y,\text{MV}\right)=\{\begin{array}{cc} 3, & \text{if}\;\;\left|{\bar{V}}_{\text{rr}}\right|=0\\ 2, & \text{else}\;\;\text{if}\;\;\left|\vec{V}{}_{s}\right|\geq \left|{\bar{V}}_{\text{rr}}\right|\\ T_{2}\left(x,y,\text{MV}\right) & \text{else}. \end{array} \end{array}$$

In formula (3), ${\bar{V}}_{\text{rr}}$ is the averaged MV in *C*_rr_; it is defined as

(4) $$\begin{array}{c} {\bar{V}}_{\text{rr}}=\frac{\sum_{\in C_{\text{rr}}}{\vec{v}}_{\text{rr}}}{\text{nu}{\text{m}}_{C_{\text{rr}}}}. \end{array}$$

Here, ${\vec{v}}_{\text{rr}}$ is the MV of MB in *C*_rr_, num_*C*_rr__ is the summation times. (*x*, *y*) denotes the position coordinates of current encoding MB.

If $\left|{\bar{V}}_{\text{rr}}\right|=0$, consider ${\vec{V}}_{s}$ is caused by MV noise and should be filtered out. ${\vec{V}}_{s}$ is set as 0 and mark the current encoding block as 3, *T*_1_(*x*, *y*, MV) = 3.

If $\left|\vec{V}{}_{s}\right|\geq \left|{\bar{V}}_{\text{rr}}\right|$, there exist obvious motion characteristics in the current encoding MB compared with the MBs in *C*_rr_, and the current encoding block belongs to dynamic foreground region which should be marked as 2, *T*_1_(*x*, *y*, MV) = 2.

Else, it means the current encoding MB has similar motion characteristics as its nearby MBs in *C*_rr_, and the current encoding block's temporal saliency characteristics are undetermined. So the translational MV checking should be carried out further in order to distinguish whether current encoding MB belongs to background region or foreground translation region.

#### 2.1.2. Translational MV Checking

After MV noise filtering procedure, the translation MV interference attenuating step comes into consideration.

(*1) Basic Principle of Translational MV Checking.* In video coding based on the block matching, first step is to get the difference value between the best matching block and the original block, namely, the prediction error. If the prediction error value is smaller, after discrete cosine transform (DCT) transform the high frequency coefficients is less, then appearing probability of all zero quantized coefficients will be higher, and when number of coding bits is fewer, the higher the compression will be, which means the current block and the predicted block has more matching higher structural similarity, and the prediction coding effect is better.

In H.264/AVC standard, if one takes 4 × 4 subblock coding as example, the integer DCT can be described as

(5) Y=(CfXCfT)⊗E=([111122−2−21−1−111−22−1][X][121111−1−21−1−121−21−1]) ⊗[a2ab2a2ab2ab2b24ab2b24a2ab2a2ab2ab2b24ab2b24],

where *C*_*f*_ *XC*_*f*_^*T*^ is integer core transform, *E* is the constant scaling matrix, and ⊗ represents matrix multiplication.

For a residual value of prediction error for 4 × 4 subblock *e*(*m*, *n*), 0 ≤ *m*, *n* ≤ 3, its transformation coefficient is *E*(*u*, *v*) computational formula is as follows:

(6) E(u,v) =∑m,n=03,3e(m,n)·[2.5C(u)2×cos⁡(2m+1)uπ8]  ·[2.5C(v)2×cos⁡(2n+1)vπ8].

In formula (6), if *u*, *v* = 0, $C(u),C(v)=1/\sqrt{2}$. If *u*, *v* ≠ 0, *C*(*u*), *C*(*v*) = 1. [·] represents the rounding computation.

After getting *E*(*u*, *v*), the computational formula of quantization coefficient *Z*(*u*, *v*) is as follows:

(7) $$\begin{array}{c} \left|Z\left(u,v\right)\right|\;=\left(\left|E\left(u,v\right)\right|\times \text{MF}\left(u,v,\text{QP}\right)+f\right)\gg q\text{bits}\\ \text{Sign}\left(Z\left(u,v\right)\right)=\text{Sign}\left(E\left(u,v\right)\right)\\ q\text{bits}=15+\text{floor}\left(\frac{\text{QP}}{6}\right). \end{array}$$

Here, MF(*u*, *v*, QP) is the multiplier factor associated with QP. ≫ stands for binary right shift operation. *f* = 2^*q*bits^/*n*, *n* = 3 or 6, corresponding intraframe coding block and interframe coding block, respectively.

If a quantized coefficient *Z*(*u*, *v*) of an encoding block's *E*(*u*, *v*) is equal to zero, it should be satisfied with the following conditions:

(8) $$\begin{array}{c} \left|E\left(u,v\right)\right|<\left|\frac{2^{q\text{bits}}-f}{\text{MF}\left(u,v,\text{QP}\right)}\right|. \end{array}$$

The sum of absolute difference (SAD) of the 4 × 4 subblock can be obtained by adding prediction error absolute value of each region. Let

(9) $$\begin{array}{c} \text{SAD}=\overset{3,3}{\underset{m,n=0}{\sum }}\left|e\left(x,y\right)\right|. \end{array}$$

So in the video coding standards based on the block-matching method, SAD is commonly used as the related function to measure the degree of correlation between the current encoding block and prediction block. The smaller value of SAD means there exiting stronger correlation between the two blocks, and they are more matchable. In this paper, the foreground translational region detection is on the basis of the of pixel region change detection theory [12].

(*2) Translational MV Checking Formula.* Let

(10) $$\begin{array}{c} T_{2}\left(x,y,\text{MV}\right)=\{\begin{array}{cc} 1, & \text{if}\;\;\text{SA}{\text{D}}_{\left(x,y\right)}\geq {\bar{\text{SAD}}}_{S_{c}},\\ 0, & \text{else}. \end{array} \end{array}$$

In formula (10), (*x*, *y*) represents the position coordinates of the encoding block. SAD_(*x*,*y*)_ is the sum of absolute difference of the current encoding block and its corresponding encoded block with the same position coordinates in previous frame. The value of SAD_(*x*,*y*)_ can be described as variation degree of encoding blocks in two adjacent video frames. SAD_(*x*,*y*)_ can be defined as follows:

(11) $$\begin{array}{c} \text{SA}{\text{D}}_{\left(x,y\right)}=\overset{M}{\underset{i=1}{\sum }}\overset{N}{\underset{j=1}{\sum }}\left|s\left(i,j\right)-c\left(i,j\right)\right|. \end{array}$$

Here, *s*(*i*, *j*) is the pixel value of the current encoding block. *c*(*i*, *j*) is the pixel value of the corresponding block in previous frame. *M*, *N* denote the partition dimensions of current encoding block, respectively.

If the value of SAD_(*x*,*y*)_ is high, it means that a great difference exists between the two adjacent frames. The current encoding block is considered in the foreground translational region under dynamic background condition and *T*_2_(*x*, *y*, MV) should be marked as 1.

If the value of SAD_(*x*,*y*)_ is low, it means that a smaller difference exists between the two adjacent frames. And the current encoding block is considered in the background region and *T*_2_(*x*, *y*, MV) should be marked as 0.

(*3) Setting of Self-Adaptive Dynamic Threshold* ${\bar{SAD}}_{S_{c}}$. As there exists diversified motion degree in video sequences, different encoding parameters, especially quantization steps, can affect the code distortion and cause change in the value of SAD_(*x*,*y*)_. How to measure SAD_(*x*,*y*)_ value becomes one of the important factors affecting the performance of the proposed algorithm. Obviously using the fixed threshold will bring judgment error. In order to reduce the detection error caused by these uncertainties mentioned above, the proposed algorithm performs translational MV interference detection with a self-adaptive dynamic threshold ${\bar{\text{SAD}}}_{S_{c}}$, which can be determined by using the averaged SAD_(*x*,*y*)_ value of all the encoding blocks in the background region in the previous frame. Let

(12) $$\begin{array}{c} {\bar{\text{SAD}}}_{S_{c}}=\frac{\sum_{(x,y)\in S_{c}}\text{SA}{\text{D}}_{\left(x,y\right)}}{\text{nu}{\text{m}}_{S_{c}}}. \end{array}$$

Here, *S*_*c*_ represents the background region in the previous frame. ∑_(*x*,*y*)∈*S*_*c*__SAD_(*x*,*y*)_ is the summation of all the SAD_(*x*,*y*)_ values for the encoding blocks enclosed in *S*_*c*_. num_*S*_*c*__ is the summation times.

(*4) Temporal Saliency Flowchart*. In summary, the computational formula of temporal saliency analysis and detection is as follows:

(13) $$\begin{array}{c} T\left(x,y,\text{MV}\right)=\{\begin{array}{cc} 3, & \text{if}\;\;\left|{\bar{V}}_{\text{rr}}\right|=0\\ 2, & \text{else}\;\;\text{if}\;\;\left|\vec{V}{}_{s}\right|\geq \left|{\bar{V}}_{\text{rr}}\right|\\ 1, & \text{else}\;\;\text{if}\;\;\text{SA}{\text{D}}_{\left(x,y\right)}\geq {\bar{\text{SAD}}}_{S_{c}}\\ 0, & \text{else}, \end{array} \end{array}$$

where *T*(*x*, *y*, MV) = 3 means the current MV is MV noise;  *T*(*x*, *y*, MV) = 2 means the current encoding block is belongs to the foreground dynamic region;  *T*(*x*, *y*, MV) = 1 means the current encoding block is belongs to the foreground translational region; and  *T*(*x*, *y*, MV) = 0 means the current encoding block is in the background region.

It should be pointed out that after filtering out the MV noise, *T*(*x*, *y*, MV) = 3, the current ${\vec{V}}_{s}$ would be set to zero; the current encoding block belongs to background region, which should be marked with 0.

**Figure 10:**
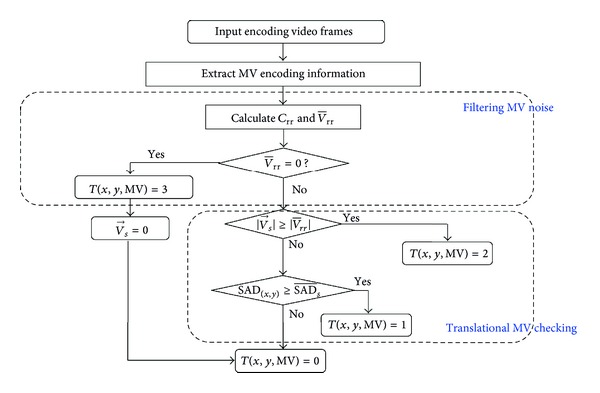
Flow chart of temporal saliency detection.

The flow chart of temporal saliency detection is shown in Figure 10.

**Figure 11:**
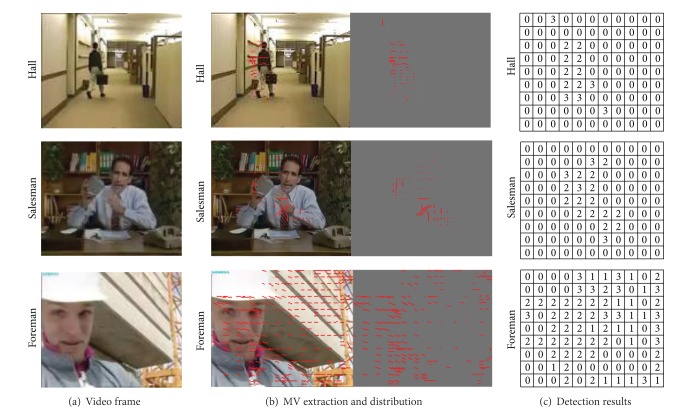
Schematic diagram of temporal saliency detection results based on MV.

According to the calculation procedure mentioned above, the current encoding frame can be sorted into temporal visual characteristic regions with different significance, based on the low-level encoding information ${\vec{V}}_{s}$ of current encoding block and its motion vector relativity with adjacent blocks in *C*_rr_ (shown in Figure 11).

As the calculation of SAD_(*x*,*y*)_ should be performed in interframe prediction mode decision and motion estimation, no additional calculation cost will be caused with the adoption of this method, so it is quite applicable in occasions with limited calculation resources.

### 2.2. Spatial Saliency Analysis and Detection

Because HVS is also sensitive to the change of spatial domain, in order to improve the visual perception of the analysis results, it needs to detect spatial saliency; analysis of the correlation between prediction mode and spatial visual features should also be performed. In the proposed algorithm, we take H.264/AVC standard as an example and discuss the correlation between prediction mode and visual spatial attention.

**Figure 12:**
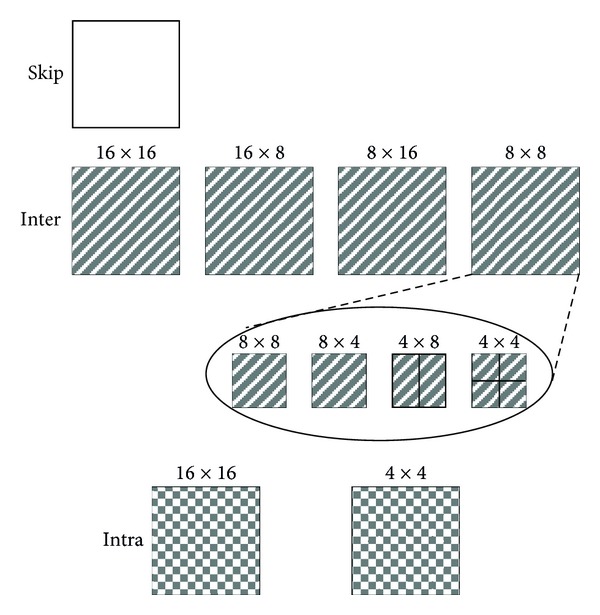
Prediction modes in H.264/AVC.

All the prediction modes of H.264/AVC coder are shown in Figure 12.

It has been verified in previous studies that the optimal prediction mode decision has the following rules [13].

In I-frame encoding of H.264/AVC standard, the smooth regions are suitable for using intra 16 × 16, while regions with rich texture always select Intra 4 × 4.

In P-frame encoding, the optimal prediction mode selection depends on the matching degree of encoded MB in forecasting, and the prediction mode selection results can describe the encoded MB's content richness and are consistent with the human visual selective attention.

As HVS is relatively nonsensitive to smooth background region, in H.264/AVC standard, the smooth regions usually choose the Intra 16 × 16 in I frame encoding or use macro-block prediction mode Inter 16 (skip, 16 × 16, 16 × 8, 8 × 16) in P frame encoding.

**Figure 13:**
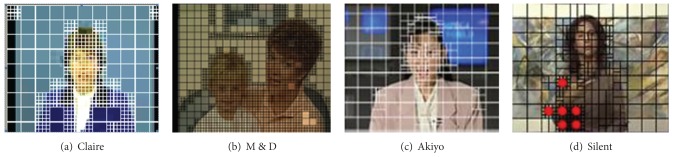
Relationship between prediction modes with visual attention. ((a), (b)) I frame encoding, ((c), (d)) P frame encoding.

HVS usually assigns higher visual importance to figures in foreground regions with abundant texture features and moving objects; therefore, those regions mentioned always select Intra 4 × 4 in I frame encoding or use subblock prediction mode Inter 8 (8 × 8, 8 × 4, 4 × 8, 4 × 4) in P frame encoding (shown in Figure 13).

Although it is small probability event that using Intra mode in P-frame coding, once Intra mode is selected, means there appears new information or the encoding content is varied greatly in current frame compared with previous frame. It can be found that, in Figure 13(d), there are 6 MBs (with red dots mark) which select Intra 4 × 4 as the optimal prediction mode; because the woman raised right arm suddenly, the moving arm is just the ROI with higher human visual attention.

There is high consistency between prediction mode decision results and visual attention. Therefore, in the proposed algorithm, the prediction mode is regarded as spatial characteristic of visual perception analysis.

According to the analysis above, current encoding frame can be sorted into spatial visual characteristic regions with different significance according to optimal prediction mode decision results. The spatial saliency detection computational formula is as follows:

(14) S(x,y,Mode) ={2,ModeP∈{Intra}1,ModeP∈{Inter 8}  or  ModeI∈Intra  4×40,ModeP∈{Inter 16}  or  ModeI∈Intra 16×16.

Mode_*P*_, Mode_*I*_ represent the optimal prediction modes selected by the current MB in P-frame and I-frame coding, respectively.

If Mode_*P*_ ∈ {Intra}, means in P-frame coding, then Intra 4 × 4 or Intra 16 × 16 is selected as the optimal prediction mode, the spatial saliency is high, the current encoding block belongs to the human visual sensitive region, and it can be expressed as *S*(*x*, *y*, Mode) = 2.

If Mode_*P*_ ∈ {Inter  8} or Mode_*I*_ ∈ Intra  4 × 4, which means the current encoding block takes the subblock prediction mode (8 × 8, 8 × 4, 4 × 8, 4 × 4) as the optimal prediction mode in P-frame coding or uses Intra  4 × 4 in I-frame coding, then the current encoding block has abundance of texture feature or changes in spatial domain, the current block belongs to attention region, the spatial saliency is high, and then it can be expressed as *S*(*x*, *y*, Mode) = 1.

If Mode_*P*_ ∈ {Inter  16} or Mode_*I*_ ∈ Intra  16 × 16, which means the current encoding block is smooth and belong to the visual nonsensitive region, then the spatial changes are slight, the current block has low spatial visual characteristics significance, it belongs to nonsignificant region, and it can be signed with *S*(*x*, *y*, Mode) = 0.

### 2.3. Combination of the Spatiotemporal Saliency Detection

In this paper, according to the spatiotemporal saliency detection results, we define video region of interest (VROI). Let

(15) $$\begin{array}{c} \text{VROI}\left(x,y,\text{MV},\text{Mode}\right)=T\left(x,y,\text{MV}\right)||S\left(x,y,\text{Mode}\right). \end{array}$$

The computational formula can be expressed as

(16) VROI(x,y,MV,Mode)  ={5, S(x,y,Mode)=24, T(x,y,MV)=2||S(x,y,Mode)=13, T(x,y,MV)=1||S(x,y,Mode)=12, (T(x,y,MV)=2  or  T(x,y,MV)=1)||S(x,y,Mode)=01, (T(x,y,MV)=0  or  T(x,y,MV)=3)||S(x,y,Mode)=10, (T(x,y,MV)=0  or  T(x,y,MV)=3)||S(x,y,Mode)=0.

In this algorithm, according to the calculation number of *T*(*x*, *y*, MV) and *S*(*x*, *y*, Mode), the priority level of VROI is divided into 6 grades, from high to low being 5~0. For example, if the current MB uses Intra mode in P-frame coding (*S*(*x*, *y*, Mode) = 2), this means that the MB is in the visual sensitive region and has the highest visual attention; it can be signed with VRIO(*x*, *y*, MV, Mode) = 5, and so on.

**Figure 14:**
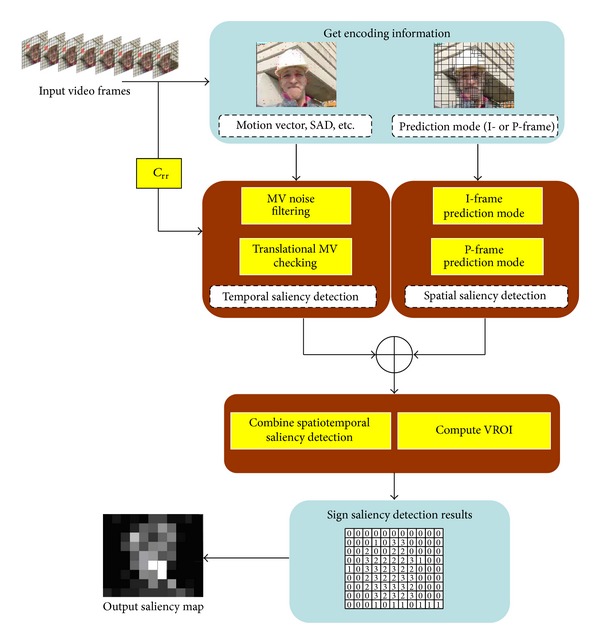
Diagram of proposed saliency detection algorithm framework.

The proposed algorithm framework is as depicted in Figure 14.

## 3. The Algorithm Performance Evaluation

### 3.1. Test Platform

In this paper, three existing algorithms are used to do the comparison experiment [4, 6, 7]. The experimental environment is set as Table 5.

We use 10 typical test sequences in multiple formats which include various types (such as 176 × 144, 352 × 288, and 416 × 240) of video with different scenes, motion, and flatness, separately, such as videos in daytime and nighttime, sports videos, news television, broadcast, and video surveillance.

### 3.2. Saliency Detection Results

**Figure 15:**
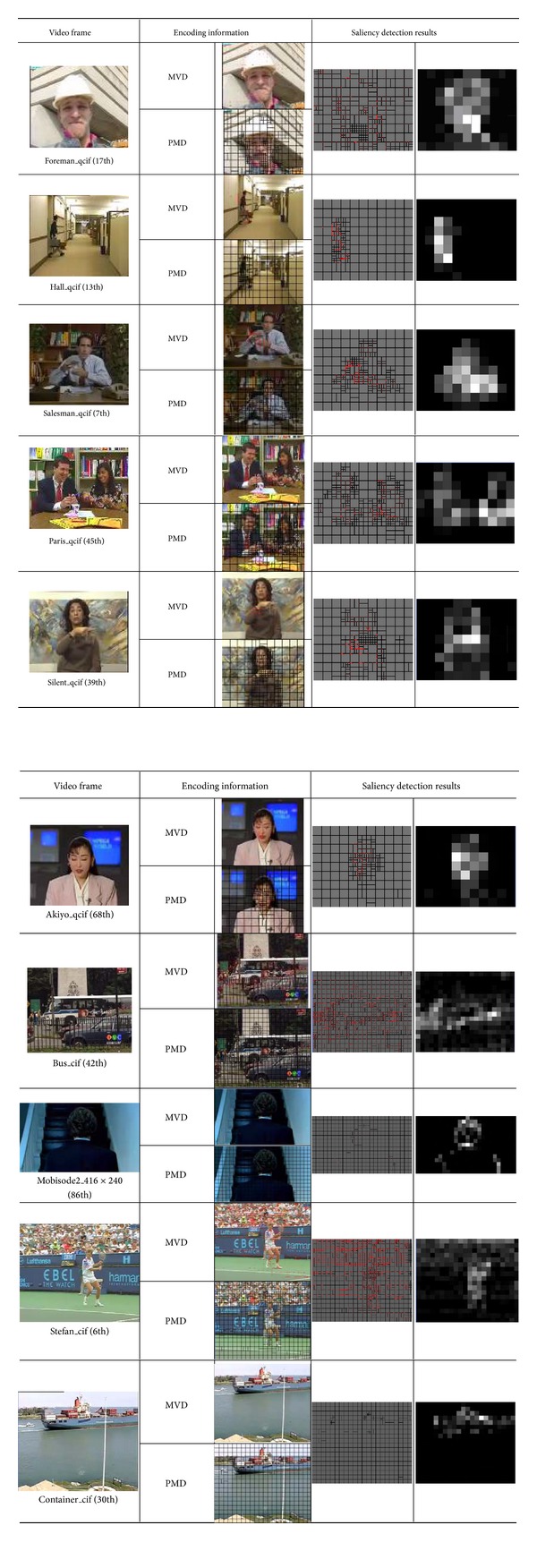
The saliency detection results of the proposed algorithm.

According to the visual perception characteristics analysis and the saliency detection procedure mentioned previously, the VROI marking results are shown in Figure 15.

In the output saliency detection results, the MB luminance values are proportional to the priority level of VRIO(*x*, *y*, MV, Mode). The region with higher visual sensitive, the corresponding MB luminance value is higher. In Figure 15, MVD is the motion vector diagram, and PMD is the prediction mode diagram. The detection results have good consistency with human visual system.

### 3.3. Algorithm Complexity Analysis

The computation time and the similar measure method are adopted to evaluate the performance of the algorithm [13]. In the similar measure method, Kullback-Leibler (KL) distance is used to measure the similarity between the saliency distributions at human saccade locations and random locations as

(17) $$\begin{array}{c} \text{KL}\left(H,R\right)=\frac{\sum_{k}h_{k}\log\left(h_{k}/r_{k}\right)+\sum_{k}r_{k}\log\left(r_{k}/h_{k}\right)}{2}. \end{array}$$

Here *H* and *R* are saliency distributions at human saccade locations and random locations with probability density functions *h*_*k*_ and *r*_*k*_, respectively.

The saliency detection algorithm with the higher KL distance can discriminate human saccade locations from the random locations more easily, and this means better performance in saliency detection for videos [14].

Statistical data in Table 6 show that the proposed algorithm can enhance the timeliness of calculation and the performance in video saliency detection markedly. Compared with [4, 6, 7], the calculation time for VROI detection can be saved up to 10.69%, 14.66%, and 5.29%; at the same time, the KL distance is increased by 0.50, 1.02, and 0.24, respectively. Especially for Bus, Stefan, Foreman, and Paris sequences, which contain a large number of global motion vectors or rich texture of background, the proposed algorithm can analyze visual perception characteristics and extraction VROI fast and accurately.

In Table 6, mathematical symbol “−” denotes decrease and “+” denotes increase. Δ*C* Time (%), ΔKL*D* are defined as follows formula in (18) and (19), respectively:

(18) ΔCTime(%) =ComputingTimecompared−ComputingTimeproposedComputingTimecompared  ×100%,

(19) $$\begin{array}{c} \Delta \text{KL}D=\text{KLDistanc}{\text{e}}_{\text{proposed}}-\text{KLDistanc}{\text{e}}_{\text{compared}}. \end{array}$$

In Table 6, the average computational time is the shortest, and the average KL distance of the proposed algorithm is the largest. This means the proposed algorithm can control the computational complexity strictly and discriminate human saccade locations from random locations more quickly and accurately than the other ones. The experiment results demonstrate that the performance of the proposed algorithm is the best among these compared ones in video saliency detection.

## 4. Conclusion

In this paper, the interdependency between video encoding information and HVS characteristics is studied; it proposes a video saliency detection algorithm based on visual perception characteristics analysis and low-layer encoding information which can get from the bit-stream directly. The simulation results show that the proposed algorithm has better performance than other existing ones. It can filter out the motion vector noise, weaken the interference of translational motion vector and get rid of visual redundancy, and it can be used in the detection of visual perception characteristics analysis and saliency detection fast and effectively. The complication of the proposed algorithm is low, and its detection results are more consistent with HVS compared with other existing algorithms. It can be used conveniently in many Internet-based multimedia applications such as video retrieval based on ROI and video quality assessment. It can also be applied to video coding standards, such as HEVC and H.264/AVC.

In the future, the various multimedia applications of the proposed video saliency detection algorithm combined with fast video coding technologies can realize fast video coding based on HVS for the latest video coding standard HEVC, and saliency detection technique can be taken as part of the video standard codec at medium-to-low bit-rates.

## Figures and Tables

**Table 1:** Probability of MV with the same position.

Video sequence	QP = 28			QP = 32		
	$\vec{V_{c}}$	$\vec{V_{s}}$	$p(\vec{V_{s}}|\vec{V_{c}})$	$\vec{V_{c}}$	$\vec{V_{s}}$	$p(\vec{V_{s}}|\vec{V_{c}})$
Akiyo	*0 *	*0 *	66.38%	*0 *	*0 *	69.81%
		*≠*0	33.62%		*≠*0	30.19%
	*≠*0	*0 *	11.92%	*≠*0	*0 *	17.35%
		$\in \vec{V_{c}}$ (1 ± 10%)	76.42%		$\in \vec{V_{c}}$ (1 ± 10%)	78.06%
		Other values	11.66%		Other values	4.59%

Foreman	*0 *	*0 *	60.86%	*0 *	*0 *	64.72%
		*≠*0	39.14%		*≠*0	35.28%
	*≠*0	*0 *	18.17%	*≠*0	*0 *	19.69%
		$\in \vec{V_{c}}$ (1 ± 10%)	65.70%		$\in \vec{V_{c}}$ (1 ± 10%)	68.02%
		Other values	16.13%		Other values	12.29%

**Table 2:** Position coordinates of vertex macroblocks with horizontal motion.

Macro blocks	Position coordinates
MB-3	*c*(*x* − *i*_*c*_, *y* − *i*_*c*_)
MB-4	*c*(*x* + *i*_*c*_, *y* − *i*_*c*_)
MB-5	*c*(*x* + *i*_*c*_, *y*)
MB-6	*c*(*x* − *i*_*c*_, *y*)
MB-7	*c*(*x* − *i*_*c*_, *y* + *i*_*c*_)
MB-8	*c*(*x* + *i*_*c*_, *y* + *i*_*c*_)

**Table 3:** Position coordinates of vertex macroblock with vertical motion.

Macro blocks	Position coordinates
MB-3	*c*(*x* − *j*_*c*_, *y* − *j*_*c*_)
MB-4	*c*(*x* − *j*_*c*_, *y* + *j*_*c*_)
MB-5	*c*(*x*, *y* + *j*_*c*_)
MB-6	*c*(*x*, *y* − *j*_*c*_)
MB-7	*c*(*x* + *j*_*c*_, *y* − *j*_*c*_)
MB-8	*c*(*x* + *j*_*c*_, *y* + *j*_*c*_)

**Table 4:** Position coordinates of vertex macroblock with oblique motion.

Macro blocks	Position coordinates
MB-1	*c*(*x*, *y*)
MB-2	*c*(*x*, *y* − *i*_*c*_)
MB-3	*c*(*x* + *j*_*c*_, *y*)
MB-4	*c*(*x* + *j*_*c*_, *y* − *i*_*c*_)
MB-5	*c*(*x*_*c*_, *y* + *i*_*c*_)
MB-6	*c*(*x*_*c*_ − *j*_*c*_, *y*_*c*_)
MB-7	*c*(*x*_*c*_ − *j*_*c*_, *y*_*c*_ + *i*_*c*_)
MB-8	*c*(*x* − *j*_*c*_, *y* − *i*_*c*_)
MB-9	*c*(*x* + *j*_*c*_, *y* + *i*_*c*_)

**Table 5:** Experimental environment.

Computer hardware	P4 @ 1.6 GHz and 2 G RAM	

Experimental software platform	H.264/AVC (JM18.7), Visual C++, Windows 2003	

Experimental parameters		
Encoding frames	Frame rate	Gop

100	30 f/s	IPPP
Entropy encoding type	QP	Search range
CAVLC	32	±16 pixels
Reference frames number	Hadamard transform	RDO
5	On	On

**Table 6:** Algorithms performance comparison results.

Sequences	Comparison algorithms	Δ*C* time (%)	ΔKL*D*
Foreman	[4]	−11.14	+0.59
	[6]	−12.88	+1.02
	[7]	−6.91	+0.32

Hall	[4]	−10.22	+0.31
	[6]	−13.75	+0.68
	[7]	−6.55	+0.21

Salesman	[4]	−12.25	+0.47
	[6]	−17.24	+0.96
	[7]	−7.12	+0.36

Paris	[4]	−12.26	+0.71
	[6]	−18.77	+1.28
	[7]	−4.90	+0.16

Silent	[4]	−7.34	+0.33
	[6]	−12.45	+0.60
	[7]	−2.76	+0.19

Akiyo	[4]	−8.19	+0.94
	[6]	−10.76	+1.56
	[7]	−4.81	+0.37

Bus	[4]	−9.75	+0.38
	[6]	−20.01	+1.49
	[7]	−5.47	+0.27

Mobisode2	[4]	−14.21	+0.31
	[6]	−10.78	+0.47
	[7]	−4.07	+0.05

Stefan	[4]	−13.38	+0.72
	[6]	−19.65	+1.34
	[7]	−5.78	+0.36

Container	[4]	−8.11	+0.22
	[6]	−10.27	+0.78
	[7]	−4.56	+0.11

Average	[4]	ΔComputing time (%)	ΔKL distance
		−10.69	+0.50
	[6]	ΔComputing time (%)	ΔKL distance
		−14.66	+1.02
	[7]	ΔComputing time (%)	ΔKL distance
		−5.29	+0.24
